# Arrhythmogenic Cardiomyopathy: the Guilty Party in Adipogenesis

**DOI:** 10.1007/s12265-017-9767-8

**Published:** 2017-10-05

**Authors:** Ilaria Stadiotti, Valentina Catto, Michela Casella, Claudio Tondo, Giulio Pompilio, Elena Sommariva

**Affiliations:** 1 Vascular Biology and Regenerative Medicine Unit, Centro Cardiologico Monzino-IRCCS, via Parea 4, 20138 Milan, Italy; 2 Cardiac Arrhythmia Research Centre, Centro Cardiolologico Monzino-IRCCS, Milan, Italy; 3 Department of Clinical Sciences and Community Health, Università degli Studi di Milano, Milan, Italy

**Keywords:** Arrhythmogenic cardiomyopathy, ARVC, Adipogenesis, Progenitors, Mesenchymal cells, Cardiomyocytes

## Abstract

Arrhythmogenic cardiomyopathy (ACM) is a genetic cardiac condition characterized by the replacement of the ventricular myocardium with fibro-fatty tissue, by arrhythmias and sudden death. Adipogenesis in ACM is considered an aberrant remodeling following myocardial loss. Which cell type(s) is (are) responsible for the adipose replacement is still matter of debate. A systematic overview of the different cells that have been, over time, considered as main players in adipose replacement is provided. The comprehension of the cellular component giving rise to arrhythmogenic cardiomyopathy substrate defects may represent both an essential tool for mechanistic studies of disease pathogenesis and a novel possible therapeutic target.

## Introduction

Arrhythmogenic cardiomyopathy (ACM) is a genetic severe cardiac condition predominantly affecting the right ventricle with unfavorable prognosis due to malignant ventricular arrhythmias and heart failure (HF). The pathological hallmark of the disease is a progressive loss of contractile myocardium, that is replaced by fibrous and adipose tissue [1]. This fibro-fatty substitution process extends transmurally with an epi-endocardial gradient, causing ventricular free wall thinning and aneurysmal dilation, especially in the so-called triangle of dysplasia (inflow tract, outflow tract, and apex) [2] of the right ventricle. Fibro-fatty substitution constitutes a part of the substrate that determines the worsening of the arrhythmogenic phenotype [3, 4]. ACM is characterized by typical electrocardiographic features (ε waves, right precordial QRS prolongation, T-wave inversion) and life-threatening ventricular arrhythmias, usually with a left bundle branch block [5]. Moreover, fibro-fatty substitution provokes progressive regional and global biventricular dysfunction [6] and ultimately congestive heart failure.

*PKP2* and the other desmosomal genes are frequently mutated in ACM [7], causing alterations of intermediate filament-mediated anchorage of cardiac cells to each other, impairing tissue structural integrity, electrical conduction, mechanical contraction, and cellular functionality [8]. To date, enough evidence has been provided indicating that electrical instability in ACM is caused by cardiomyocyte defects, including dysregulation of sodium channels, gap junctions, and intracellular calcium handling processes, and ultimately by cardiomyocyte death [4]. On the contrary, adipose replacement, which is likely to be attributed to a defective remodeling following myocardial loss [9], lacks an indisputable proof about its origin.

Cardiomyocyte loss has been attributed to different pathogenic mechanisms, among which stretch-induced cell damage [10] facilitated by desmosomal mutations. Moreover, since inflammatory infiltrates have been found in ACM hearts, the co-occurrence of myocarditis has been postulated to further produce cardiomyocyte injury and death [11]. Finally, the myocyte apoptotic theory has been formulated due to the high levels of CPP-32, a cysteine protease required for apoptosis, detected in ACM patients’ hearts [12]. Whatever the reason, cardiomyocytes die and subsequently fibro-fatty substitution occurs.

The “infiltrative theory” of cells from the epicardial layer [13] has been advanced as a plausible hypothesis for fibro-fatty substitution, according to evidence at both tissue and clinical levels that fibro-fatty infiltration progresses from the epicardium towards the endocardium [14].

On the contrary, the “differentiation theory” proposes that resident cardiac cells differentiate into adipocytes, through Wnt pathway alterations. Indeed, it was hypothesized that an altered desmosomal structure could provoke plakoglobin (PG) translocation to the nucleus, where it could induce cell transcriptional activity changes. This could lead to the increase of adipogenic and fibrogenic gene expression, thus contributing to differentiation [15]. However, PG nuclear translocation, demonstrated *in vitro* by different groups [15–18], has been questioned in human pathological ACM heart tissues [19].

Recently, the Hippo pathway, activated by mechanical stress, G-protein-coupled receptor signaling, and oxidative stress [20], has been found involved, with the effect of dysregulating cell proliferation/apoptosis and further suppressing Wnt pathway [21].

Although both theories are supported by a strong rationale, so far a single theory has not been exclusively proven by clear-cut evidence. Which cell type(s) is (are) responsible for the aberrant adipogenic differentiation process is therefore still an open and burning question. This review aims to provide a systematic overview about possible cell effectors that have been proposed as players of adipose replacement in ACM and is meant to provide a new vision of the mechanisms of fibro-fatty development, in an attempt to harmonize the “infiltrative” and “differentiation” theories.

## Mechanisms of Adipogenesis

The comprehension of molecular mechanisms underpinning adipose tissue development is crucial to characterize the presence of adipocytes in ACM hearts. This phenomenon in ACM occurs through a proper adipogenic differentiation process producing mature adipocytes (adipogenesis), and not mere cellular lipid accumulation (lipogenesis).

**Fig. 1:**
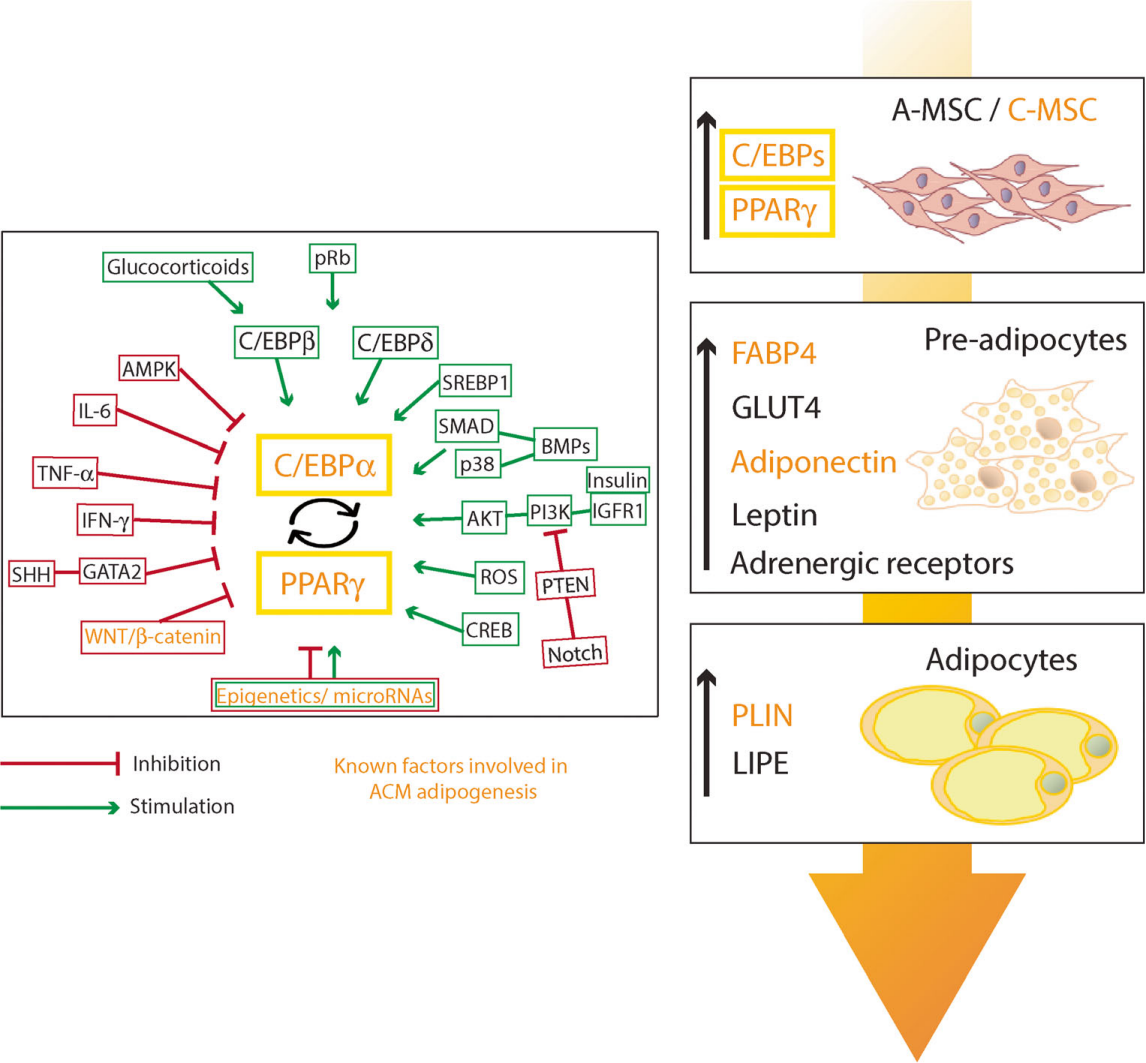
Common mechanisms of adipogenic differentiation in adipose tissue and in arrhythmogenic cardiomyopathy (ACM) hearts. The differentiation of adipose tissue-mesenchymal stromal cells (A-MSC) to adipocytes is driven by different signaling pathways, transcription factors, and epigenetic mechanisms. The box on the left highlights the complex regulation of adipogenesis, including the mechanisms responsible of inhibition (in *red*) and stimulation (in *green*) of adipocyte formation. In the right part of the figure, transcript upregulation during A-MSC differentiation into preadipocytes and then adipocytes is illustrated. The *orange writing* represents the cell source (cardiac mesenchymal stromal cells (C-MSC)), and the mechanisms of adipogenesis demonstrated in ACM. *AKT* protein kinase B, *AMPK* AMP-activated protein kinase, *BMPs* bone morphogenetic proteins, *CEBPs* CCAAT/enhancer-binding proteins, *CREB* cAMP responsive element binding protein, *FABP4* fatty acid-binding protein 4, *GATA2* GATA binding protein 2, *GLUT4* glucose transporter type 4, *IFN-γ* interferon-γ, *IGFR-1* insulin-like growth factor receptor, *IL-6* interleukin, *LIPE* lipase, *p38* protein 38, *PI3K* phosphatidyl inositol 3-kinase, *PLIN* perilipin, *PPARγ* peroxisome proliferator-activated receptor-γ, *pRB* retinoblastoma proteins, *PTEN* phosphatase and tensin homolog, *ROS* reactive oxygen species, *SHH* sonic hedgehog, *SREBP1* sterol regulatory element binding protein 1, *TNF-α* tumor necrosis factor-alpha

In adipose tissue, adipocyte formation is a multifaceted and stepwise differentiation process involving many mediators (Fig. 1). Adipocytes derive from adipose tissue-mesenchymal stromal cells (A-MSC), differentiating at first into lipoblasts, then to preadipocytes and finally into mature adipocytes [22]. The acquisition of the adipocyte phenotype is characterized by sequential changes in the expression of many genes [23]. The primary factors activated by adipogenic stimuli are CCAAT/enhancer-binding proteins (C/EBPs) β and δ, that are responsible for peroxisome proliferator-activated receptor gamma (PPARγ) and C/EBP-α expression increase, in parallel with cell growth arrest [24]. PPARγ and C/EBPα mutually reinforce positive regulatory loops ensuring their high-level expression. Moreover, sterol regulatory element binding protein 1 (SREBP1) transcription factor is induced at the early stages of the adipogenic process [25].

PPARγ is the master regulator, being essential for the adipogenic process [26]. Its expression progressively increases to allow the adipogenic switch [23]. It is regulated by several known signals, among which Wnt pathway is one of the major negative mediators [27]. The canonical Wnt pathway is activated by Wnt ligands leading to the translocation of β-catenin into the nucleus, where it binds the T cell/lymphoid enhancer factor (TCF/LEF) transcription factors, stimulating proliferation genes [28]. When Wnt is inhibited, a switch occurs between proliferation and adipogenic fate.

The inhibition of sonic hedgehog (SHH) pathway triggers adipocyte differentiation, even if alone is not sufficient to succeed in [29]. SHH may induce anti-adipogenic effects by enhancing GATA binding protein 2 (GATA2) expression, leading to the inhibition of PPARγ and C/EBPα expression [30].

Bone morphogenetic proteins (BMPs), proteins of the transforming growth factor β (TGFβ) super-family, can induce adipogenesis through the activation of Smad transcription factors and p38 mitogen-activated protein kinase (MAPK) pathway [31].

AMP-activated protein kinase (AMPK) is involved in the negative regulation of adipogenesis. It blocks the expression of PPARγ and C/EBPα and promotes apoptosis [32].

When complexed with its ligands, the insulin-like growth factor receptor (IGFR)-1 can activate different downstream effectors, among which phosphatidyl inositol 3-kinase (PI3K), leading to the activation of the serine threonine kinase Akt cascade, thus stimulating adipogenesis [33]. A link is known between PI3K and Notch signaling. In particular, phosphatase and tensin homolog (PTEN) was demonstrated to be a negative regulator of the PI3K pathway [34].

The induction of adipogenesis is promoted by glucocorticoids, through the activation of C/EBPβ [35], cAMP responsive element binding protein (CREB) through the increase of PPARγ expression [36], and retinoblastoma (pRb) family proteins through direct interaction with C/EBPs [37].

Other factors able to modulate adipogenesis are reactive oxygen species (ROS) and free radicals, that can interact with preadipocytes to prompt their differentiation [38].

On the contrary, pro-inflammatory cytokines such as tumor necrosis factor-alpha (TNF-α), interleukin (IL)-6, and interferon-γ (IFN-γ) inhibit preadipocyte differentiation and lipid accumulation [39–41].

In addition to transcription changes induced by transcription factors, also epigenetic mechanisms and microRNAs, fine regulators of gene expression, are involved in the adipogenic process [42].

At later phases, when preadipocytes have committed to the adipogenesis program, activation of metabolic genes and adipokines occurs, such as fatty acid-binding protein 4 (FABP4), glucose transporter 4 (GLUT4), leptin, and adiponectin [27]. Increased levels of total adrenergic receptors are also reported [23]. In addition, adipocytes synthesize other adipose tissue-specific products, such as perilipins, lipid droplet-surrounding proteins [43], hormone-sensitive lipase, that hydrolyzes stored triglycerides to free fatty acids [44], and the fatty acid transporter CD36 [23]. This process provokes changes in cell morphology, with conversion from fibroblastic to spherical shape, along with modifications of cytoskeletal and extracellular matrix components [23].

Analogies can be found in the differentiation process occurring in adipose tissue and in ACM hearts. As described, the involvement of certain adipogenic pathways is clearly established in ACM [3, 15, 16, 45] (Fig. 1). Indeed, as mentioned above, Wnt/β-catenin pathway and different microRNAs have been shown to ultimately affect the C/EBPα-PPARγ axis in ACM. Mutated cells upregulate adipogenic transcripts, such as FABP4 and adiponectin, and proceed to differentiation into mature PLIN-expressing adipocytes (Fig. 1). Other adipogenic mechanisms are still unexplored, even if potentially involved.

## Which Cell Is the Source of Fibro-Fatty Substitution in ACM?

### Cardiomyocyte Transdifferentiation

The hypothesis that adipocytes in ACM hearts derive from cardiomyocyte transdifferentiation has gained in the past a widespread consensus. Interestingly, a single report, published more than 15 years ago, described that the histological, immunochemical, and ultrastructural analysis of an ACM heart was suggestive of cardiomyocyte adipogenic transdifferentiation [46]. The authors found, in both ventricles, that cardiomyocytes contiguous to the adipose tissue showed, instead of the myofibril component, multiple sarcoplasmic vacuoles, that made these cells indistinguishable from preadipocytes. By means of immunohistochemistry staining for desmin to mark muscle tissue, and vimentin, a protein expressed in adipocytes and absent in adult cardiomyocytes [46], they observed that a few myocytes showing cytoplasmic vacuoles were positive for both these markers. They interpreted these cells as transitional elements between cardiomyocytes and fat cells. However, vimentin is not exclusively expressed in adipocytes, being a major cytoskeletal component of mesenchymal cells; therefore, this evidence is not *per se* demonstrative of myocyte transdifferentiation. This evidence has never been confirmed to date. In a recent work, we could not detect in human ACM hearts cardiomyocyte positivity for the adipocyte specific marker PLIN1 as well as preadipocyte positivity for α-sarcomeric actin [16]. Thus, this evidence excludes a direct contribution of adult cardiomyocyte transdifferentiation into adipocytes.

Interestingly, in 2008, Fujita et al. [47] observed in an ACM patient’s heart biopsy a large amount of adipose tissue and a group of isolated myocardial cells with an island-like appearance between the adipocytes. Analyzing this group of cardiomyocytes, they detected polymorphic nuclear changes in shape, perinuclear vacuolization, and accumulation of small granules, which visually appeared similar to lipid droplets. By means of electron microscopy, they speculated on the presence of lipid droplets of various sizes in the cardiomyocytes. This phenomenon was accompanied by degeneration of intracellular organelles and occasional disruption of the plasma membrane with discharge of intracellular content, including mitochondria, into the interstitial space [47], all being characteristics of cellular autophagy [48]. Although the microscopic analysis is not conclusive for cardiomyocyte lipid accumulation, a limited lipogenesis (fat droplet accumulation) may occur in myocytes [45]. It is then conceivable that the contractile heart compartment undergoes lipogenesis rather than adipogenesis in ACM. Of note, intracellular fat droplet accumulation has the potential to impact on myocardial function [49].

### Cardiac Progenitor Cells

As an alternative causative mechanism, cardiac progenitor cells expressing desmosomal proteins have been postulated to be responsible for adipocyte differentiation in ACM hearts.

Taking advantage of genetic fate-mapping experiments in mice, second heart field-derived progenitor cells, characterized by Isl-1, an early marker of multipotent mesodermal precursors, have been shown to take part to adipogenesis. This finding was corroborated in ACM human hearts by the evidence of co-expression of second heart field markers and adipogenic transcription factors [50]. Notably, Isl-1 cell expansion has been related to Wnt signaling [51]. On this basis, it has been suggested that the adipogenic switch of these precursors may occur before myocyte commitment, depending on the suppression of canonical Wnt pathway signaling by nuclear PG translocation [50]. However, the small number of Isl-1^+^ preadipocytes found in ACM hearts [50] is a strong limitation challenging this hypothesis. Moreover, the embryologic origin of cardiac progenitors from the second heart field cannot provide an exhaustive explanation for the left-predominant or biventricular forms of ACM [52].

A further hypothesis involving cardiac precursors is based on the common developmental origin of second heart field RV progenitors and epicardial cells [53]. Epicardial cells, giving rise to non-myocyte stromal components through epithelial-to-mesenchymal transition (EMT) [54], have been proposed as source of adipocytes in ACM [13]. Indeed, Matthes et al. demonstrated that epicardium-derived cells obtained from neonatal hearts express desmosomes, and, if silenced for *PKP2*, they have increased migration velocity, higher proliferative rate, and can be driven to adipogenesis. These cells have been in fact suggested to cause excess of fibroblasts, adipocytes, or their progenitors, in the myocardial interstitium [13]. As an E-cadherin negative and α-smooth muscle actin (α-sma) positive subpopulation was identified, the authors hypothesized that this epicardial cell subpopulation may correspond to resident myofibroblast, or alternatively cells in which EMT occurred [13]. After *PKP2* silencing, an increase in α-sma-positive cells was reported, and it was not clear whether it depended on cell proliferation or on a boost of EMT. Indeed, in EMT process, both E-cadherin and PKP2 are known to be downregulated [55], raising the intriguing hypothesis that PKP2 loss could be associated to EMT. The epicardium-endocardium gradient of the ACM adipose substitution is a key element supporting this theory [9].

Alternatively, c-kit^+^/Sca1^+^ progenitor cells have also been proposed as adipocyte precursors [17]. Lombardi et al. in 2011 generated transgenic mice overexpressing wild-type and truncated PG. Histological examination showed a moderate increased number of adipocytes, predominantly in the epicardium, and fibrosis in the heart of these transgenic models. Moreover, they exhibited cardiac dysfunction and premature death. The authors isolated c-kit^+^/Sca1^+^ cells from PG transgenic mice, and they obtained adipogenic differentiation of these cells through the contribution of Wnt signaling [17]. Since c-kit^+^/Sca1^+^ cells from PG homozygous knockout embryos have been shown to be resistant to adipogenesis and showed normal activation of canonical Wnt pathway, they demonstrated the role of PG in the induction of lipid accumulation in these cells [17]. However, a quantitative evaluation providing evidence on the effective contribution of these cardiac progenitor cells on adipogenesis is currently lacking [56]. Our recent findings revealed that the percentage of c-kit^+^ preadipocytes in the human ACM myocardium is too low to account for the massive adipocyte presence in ACM hearts, suggesting that only a small proportion of adipocytes in ACM may ultimately originate from c-kit^+^ progenitors [16].

### Differentiation of Pluripotent Cells

In 2016, we introduced for the first time a novel hypothesis on the origin of ectopic adipocytes in ACM [16], identifying cardiac mesenchymal stromal cells (C-MSC) as an adult, non-contractile cell compartment involved in lipid accumulation and adipogenic differentiation [16]. C-MSC are a large population of supportive cells of epicardial origin, characterized by multipotency. They play a critical role in maintaining healthy heart structure and function. Importantly, C-MSC are involved in cardiac remodeling in pathological conditions [57]. Noteworthy, as for A-MSC, C-MSC can differentiate into adipocytes under appropriate *stimuli*.

First of all, to understand the cellular origin of adipocytes in ACM hearts, we performed immunofluorescence staining on ACM cardiac tissue, finding cells actively differentiating into adipocytes expressing the typical mesenchymal markers CD29 and CD105 [16].

Moreover, we isolated C-MSC from ACM and control heart biopsy specimens, and we demonstrated that C-MSC express desmosomal genes, and therefore suffer the consequence of desmosomal mutations. Importantly, C-MSC from ACM patients’ hearts have been shown to differentiate into adipocytes in culture, by both phenotypic analysis and expression analysis of specific adipogenic genes and relative proteins. We also tested the involvement of the Wnt pathway, increasing its activity by inhibiting glycogen synthase kinase 3 beta (GSK3β), and obtained a reduction in the accumulation of lipid droplets. Since *PKP2* is expressed at lower levels in ACM C-MSC, not only when obtained from *PKP2* mutation carriers but also when obtained from patients without a known mutation, we evaluated if adipogenic differentiation occurs through a common *PKP2*-dependent mechanism. Indeed, the overexpression of *PKP2* led to a decrease of lipid accumulation in ACM cells. In contrast, silencing *PKP2* in control cells, we obtained a significant increase of adipogenesis. All together, these findings provide convincing evidence that C-MSC are a source of adipocytes in ACM. In the future, genotype/phenotype studies need to be performed in order to understand any differential propensity of C-MSC lipid accumulation in different desmosomal or non-desmosomal mutation carriers.

More recently, cardiac fibro-adipose progenitors (FAP) characterized by the expression of PDGFRα have been proposed as source of both adipocytes and fibrosis [58], as previously suggested by Paylor and colleagues [56] and as demonstrated by the expression of either adipogenic or fibrogenic markers. FAP progenitors contribute to the vascular and mesenchymal compartments of the human hearts and descend from epicardial derivatives, being PDGFRα necessary for EMT[59]. Lombardi et al. demonstrated that the subset of FAP that expresses the adipogenic marker C/EBP-α expresses also desmosomal genes, and, if mutated, can differentiate into adipocytes through the suppression of canonical Wnt signaling in ACM caused by *DSP* haploinsufficiency. Cardiac FAP gives origin to approximately 40% of adipocytes in the heart of an ACM mouse model, indicating the possibility of the co-existence of another cellular origin of lipid accumulation in ACM. Further experiments will be needed to ascertain communalities and differences of C-MSC [16] with FAP subpopulation [58] in ACM pathogenesis.

## Inferences and Conclusions

Research in ACM has recently progressed from clinical, genetic, and mechanistic standpoints. Pathogenic information is however still missing for a complete understanding of ACM etiology, even if scientific consensus exists on key points as follows:

1. It is now widely accepted that cardiomyocyte death constitutes the *primum movens* in disease pathogenesis, either apoptotic [12] or secondary to mechanical stretch [10] or infections [11].
2. Aberrant remodeling after cardiomyocyte death takes place through signaling pathways [15, 21] specifically driven by desmosomal gene mutations, resulting in overdeposition of extracellular matrix and desmosome-expressing cell differentiation into adipocytes.

Relevant steps forward have been undertaken over time in the understanding of fibro-fatty pathogenesis and cellular effectors (Table 1). As outlined in this manuscript, cardiomyocyte transdifferentiation is unlikely to happen. Furthermore, cardiac progenitors are present in a too low rate in an adult heart to account to a wide effect contribution. Most likely, epicardial cells, highly expressing desmosomal genes, as all epithelia, undergo EMT, of which Wnt represents a contributing pathway, resulting in a centripetal invasion of mesenchymal cells from the epicardium to the endocardium, both in ACM and in physiological conditions. C-MSC are characterized by a lower expression of desmosomal genes with respect to epithelial cells; particularly, the further reduction of desmosomal proteins in ACM C-MSC may be the culprit for the adipogenic switch. Indeed, C-MSC or a C-MSC subpopulation are well known to possess a differentiation ability into adipocytes.

**Table 1:** Summary of all cells proposed as candidate sources of adipocytes in ACM hearts

Cell	Reference	Species	Strengths	Weakness
Cardiomyocyte	D’Amati et al. [46]	Human	- Cardiomyocytes highly express desmosomal proteins.	- No demonstration of cardiomyocyte adipogenic differentiation.
	Fujita et al. [47]			- Not demonstrative of cardiomyocyte adipogenic differentiation. - Lipogenesis only.
Cardiac progenitor	Lombardi et al. [50]	Mouse	- Co-expression of second heart field markers and adipogenic transcription factors. - Is1-1^+^ cell expansion is related to Wnt signaling.	- Small number of Is1-1^+^ cells in an adult heart. - Do not explain biventricular and left forms of ACM. - Demonstrated in animal model only.
	Matthes et al. [13]	Rat	- Epicardial cells express desmosomal proteins. - If silenced for *PKP2*, increased their migration velocity, proliferative rate, and accumulate lipids.	- Small number of epicardial cells in an adult heart. - Demonstrated in animal model only.
	Lombardi et al. [17]	Mouse	- c-kit^+^/Scal^+^ cells undergo adipogenic differentiation, with the involvement of PG.	- Small number of c-kit^+^/Scal^+^ cells in an adult heart. - Demonstrated in animal model only.
Pluripotent cell	Sommariva et al. [16]	Human	- C-MSC express desmosomal proteins. - Preadipocytes in ACM hearts are of mesenchymal origin. - C-MSC undergo adipogenic differentiation with the involvement of Wnt.	- Need for further characterization.
	Lombardi et al. [58]	Human/mouse	- FAP express desmosomal proteins. - FAP undergo adipogenic differentiation with the involvement of Wnt.	- FAP are not the only responsible for ACM cardiac adipocytes. - No evidence on patients’ hearts.

The strengths and weakness of the different studies are listed.

Whether adipogenesis in ACM hearts represents distorted wound healing or has a distinct functional role in disease pathogenesis is still not known. Electrically inert tissue may induce compulsory current routes through the remaining cardiomyocytes, exacerbating ACM arrhythmic phenotype. Moreover, adipose tissue is known to exert a strong paracrine activity, influencing both inflammation and cardiomyocyte contractility performances by attenuating intracellular calcium ion levels [60].

Overall, a comprehensive understanding of the fibro-fatty substitution process in ACM is today a matter of active scientific debate with potential relevant repercussion at clinical level, since the cellular component giving rise to substrate defects may represent both an essential tool for mechanistic studies of ACM pathogenesis and a possible novel therapeutic target. Indeed, the *in vitro* study of the adipogenesis process in the cells responsible for the adipose tissue deposition in ACM may give scientists the tools to counteract it by directly involved pathways. Another interesting option may be to assess a potential therapeutic effect by means of high-throughput screenings of FDA-approved drugs or new druggable compounds. Perspectively, targeted cell-specific administration could be envisaged, in order to avoid systemic effects.

## Abbreviations

ACM: Arrhythmogenic cardiomyopathy
AMPK: AMP-activated protein kinase
A-MSC: Adipose tissue-mesenchymal stromal cells
BMP: Bone morphogenetic protein
CD36: Cluster of differentiation 36
C/EBP: CCAAT/enhancer-binding protein
C-MSC: Cardiac mesenchymal stromal cells
CREB: cAMP responsive element binding
DSP: Desmoplakin
EMT: Epithelial-to-mesenchymal transition
FABP4: Fatty acid-binding protein 4
FAP: Fibro-adipose progenitors
FDA: Food and Drug Administration
GATA2: GATA binding protein 2
GLUT4: Glucose transporter 4
GSK3β: Glycogen synthase kinase 3 beta
HF: Heart failure
IGFR-1: Insulin-like growth factor receptor
IL-6: Interleukin-6
IFN-γ: Interferon-γ
Isl-1: Insulin gene enhancer 1
MAPK: Mitogen-activated protein kinase
PDGFRα: Platelet-derived growth factor receptor α
PG: Plakoglobin
PI3K: Phosphatidyl inositol 3-kinase
PIPKB: Phosphatidyl inositol phosphate kinase B
PKP2: Plakophilin 2
PLIN: Perilipin
PPARγ: Peroxisome proliferator-activated receptor gamma
pRb: Retinoblastoma
PTEN: Phosphatase and tensin homolog
ROS: Reactive oxygen species
SHH: Sonic hedgehog
SREBP: Sterol regulatory element binding protein
TCF/LEF: T cell factor/lymphoid enhancer factor
TGFβ: Transforming growth factor β
TNF-α: Tumor necrosis factor-alpha
α-sma: α-Smooth muscle actin
